# Biochemistry of fluoroprolines: the prospect of making fluorine a bioelement

**DOI:** 10.3762/bjoc.17.40

**Published:** 2021-02-15

**Authors:** Vladimir Kubyshkin, Rebecca Davis, Nediljko Budisa

**Affiliations:** 1 Department of Chemistry, University of Manitoba, 144 Dysart Rd., Winnipeg, R3T 2N2, Canada; 2 Institute of Chemistry, Technical University of Berlin, Müller-Breslau-Str. 10, 10623 Berlin, Germany

**Keywords:** amino acids, evolution, fluorine, proline, proteins

## Abstract

Due to the heterocyclic structure and distinct conformational profile, proline is unique in the repertoire of the 20 amino acids coded into proteins. Here, we summarize the biochemical work on the replacement of proline with (4*R*)- and (4*S*)-fluoroproline as well as 4,4-difluoroproline in proteins done mainly in the last two decades. We first recapitulate the complex position and biochemical fate of proline in the biochemistry of a cell, discuss the physicochemical properties of fluoroprolines, and overview the attempts to use these amino acids as proline replacements in studies of protein production and folding. Fluorinated proline replacements are able to elevate the protein expression speed and yields and improve the thermodynamic and kinetic folding profiles of individual proteins. In this context, fluoroprolines can be viewed as useful tools in the biotechnological toolbox. As a prospect, we envision that proteome-wide proline-to-fluoroproline substitutions could be possible. We suggest a hypothetical scenario for the use of laboratory evolutionary methods with fluoroprolines as a suitable vehicle to introduce fluorine into living cells. This approach may enable creation of synthetic cells endowed with artificial biodiversity, containing fluorine as a bioelement.

## Introduction

Nature employs a rather small set of chemical elements for constructing the core biochemical makeup. Most elements of the periodic table are excluded from the biochemical world. Not surprisingly, researches have been intrigued by an idea to introduce certain elements into biochemical schemes artificially. Of the many possible elements to introduce, fluorine is arguably the most suited for being used as an artificial bioelement [1]. Organofluorine chemistry is very diverse, and many organofluorine compounds are compatible with biochemical settings [2–3]. In addition, a number of organofluorine compounds occur in the metabolism of some niche organisms [4–7].

Proteins are key molecular entities acting in cellular processes, and they serve for numerous biochemical functions, such as structural, catalytic, energetic, and transport. The introduction of organofluorine components into proteins is typically expected to alter their structure, stability and/or specific features associated with their functional roles [8–11]. The replacement of natural amino acid residues with the ones containing fluorine have been particularly well characterized for their use as spectroscopic probes, mainly in ^19^F NMR applications [12–15]. It is typical that the incorporation of fluorine into certain molecular fragments changes the local polarity as well as the electronic and conformational properties. These factors may translate into an altered structure and stability of a protein containing a fluorinated fragment. What consequences fluorination would have regarding the fitness and survival of the organism relying on fluorine-containing proteins remains an open question.

**Figure 1:**
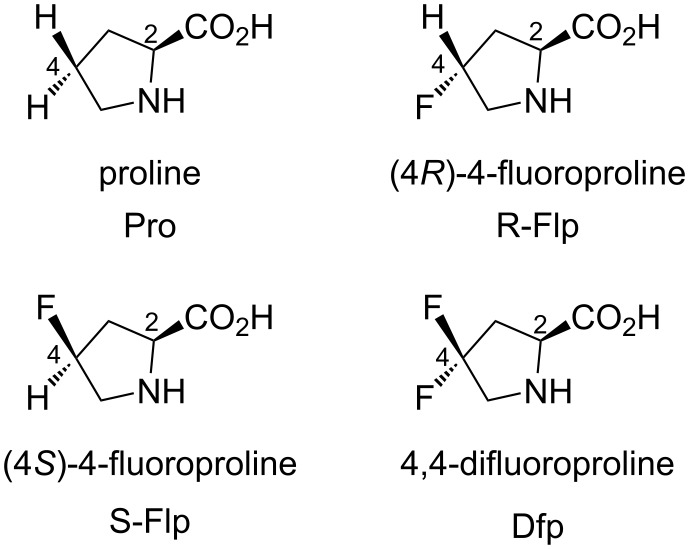
The structures of the fluoroprolines discussed herein.

In this context, the substitution of the proline residue with fluorinated analogues (fluoroprolines, Figure 1) creates an option for making fluorine a component of a living organism. Fluoroprolines were found to be generally compatible with the cellular machinery, in particular the one that transports them inside the cells and incorporates them into proteins [16]. The effects of fluoroprolines have been examined in a number of protein structures. These studies demonstrated an altered stability and altered folding kinetics that occurred upon the proline-to-fluoroproline replacement, as reviewed in [17]. Nonetheless, no whole-cell adaptation to fluoroprolines has been accomplished to date. This critical review aims to assess the potential of fluoroprolines to affect the biochemical composition of microbial organisms, with the focus on *Escherichia coli* (*E. coli*). In our analysis, we will recapitulate the canonical functions of proline in cellular biochemistry and the potential of fluoroprolines to fulfill them. Here, by fluoroprolines, we will only refer to 4-monofluoroprolines and 4,4-difluoroproline, which are the best biochemically characterized proline analogues (Figure 1). Many other fluorinated proline analogues exist, as summarized elsewhere [18–19]. They are not included in this review due to a scarcity of biochemical studies.

## Review

### 1 The special role of proline in proteins

In accord with contemporary evolution theories, a primitive proteome of protocells is thought to have developed from a small set of amino acids, with proline generally being among the first structures in this list [20]. It is possible that the initial role of proline was metabolic or based on its organocatalytic features that were essential for the development of primitive molecular life [21]. Furthermore, such an evolution model suggests that the first protein-biosynthesis-generated structures are based on variable backbone elements; proline (cyclic and chiral), glycine (acyclic and achiral) or alanine (acyclic and chiral). Subsequent acquisition of the functional side-chains for this repertoire led to the recruitment of amino acids that are essentially derived from alanine, leading to the α-helix-dominated protein world as we know it [22]. For this reason, most amino acids in proteins can be exchanged for alanine by point mutations while leaving the secondary structure intact. In molecular biology, the alanine scanning method takes advantage of the fact that alanine mimics the secondary structure preferences of most proteinogenic amino acids, allowing the relative importance of each side chain for the biological function to be determined [23]. Classical X-ray crystallography also employs the polyalanine model for backbone chain tracing to elucidate three-dimensional structures of proteins.

**Figure 2:**
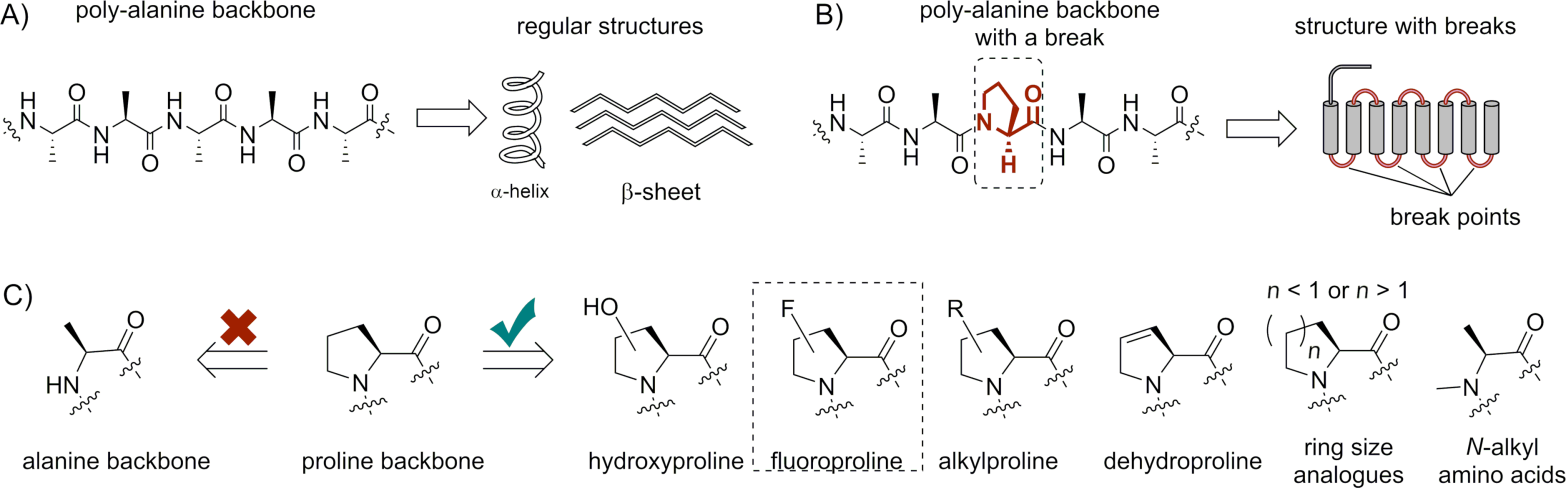
The distinction between “the alanine and the proline worlds”. While the polyalanine backbone leads to the formation of regular secondary structures (A), proline residues create breaks in it (B). C) The set of amino acids that are typically considered as proline analogues.

However, the substitution of proline by one of the remaining 19 coded amino acids can be harmful to protein folding and the functional properties. This is usually explained by the absence of the amide hydrogen atom and the cyclic nature of the proline ring, which exhibits a distinct conformational profile [24]. These are the general reasons why proline is poorly compatible with regular α-helical and β-sheet structure architectures, which are typical for the polyalanine backbone. Consequently, proline often identifies secondary structure breaks in protein sequences (Figure 2A and Figure 2B). This way of reasoning makes proline (as well as glycine) a kind of foreign structural element in the predominantly alanine-based protein architectures, or in other words, it is an alien in the “alanine world”.

How could proline be replaced in protein structures of living organisms? In protein evolution, mutations obey a simple "similar replaces similar" rule since the disruption of the resulting protein structure is minimized by the modest changes in the amino acid backbone. Hence, when global substitution of proline is aimed at, it should possibly be ascribed to its analogous structures. Most typical among proline analogues would be hydroxy-, fluoro-, alkyl-, dehydroprolines, analogues having ring size variations, and *N*-alkylamino acids (Figure 2C). To clearly discriminate the alanine and proline-based architectures, we recently proposed to call this set of structures the “proline world” [25]. Pioneering experiments on the substitution of proline by fluorinated proline analogues in proteins date back to the 1960s [26–28]. These experiments were predominantly conducted to address the role of proline residue hydroxylation in collagen.

The conformational rigidity featured in proline residues as well as the high conformational stability of the *trans*- and *cis*-amide conformers assign proline another important structural role: the structure freezer. The incorporation of proline residues in connecting loops has been described as a general strategy towards the thermodynamic stabilization of proteins [29–31]. The regulation of the proline content in proteins is an evolutionary strategy for environmental adaptation in extremophilic organisms. Proline is of a higher abundance in the proteomes of thermophilic organisms (organisms living at a high temperature) and of a lower abundance in the proteomes of psychrophilic organisms (organisms living at a cold temperature) [32–33]. In this way, the proline residue content is utilized to balance out the environmental temperature conditions.

Due to the lack of the side-chain functional groups, posttranslational modifications of proline residues are sparse. The most common among them is hydroxylation at position 4 by molecular oxygen, which is mediated by prolyl-4-hydroxylase [34]. This process has a remarkable relevance in the stabilization of collagen in higher organisms [35]. The experimental expression of the hydroxylating enzyme in *E. coli* has been previously achieved [36–37]. Bacterial prolyl hydroxylases have been characterized, and they are able to oxidize proline and analogous structures in recombinantly expressed proteins [38]. Nonetheless, we are still missing evidences of natural posttranslational modifications of proline residues occurring in native *E. coli*.

### 2 Physicochemical properties of fluoroprolines

Organofluorine compounds have become very common in various medicinal chemistry applications due to a number of special features [39–40]. Perhaps, the most notable trait among them is their stability. The carbon–fluorine bond is one of the strongest covalent bonds, and this is the strongest single bond that carbon can make. This is why molecular fragments that contain isolated C–F bonds appear inert under biochemically relevant conditions (Notwithstanding exceptions [41]). Fluoroprolines can be easily handled both as free amino acids and as residues in peptides and proteins. Nonetheless, when compared to the parent proline structure, fluoroprolines possess a number of altered properties. Some properties of the amino acids in common model compounds are summarized in Table 1 and will be discussed below.

**Table 1:** Summarized molecular features of the methyl esters of *N*-acetylamino acids as model compounds.^a^

entry	compound	COSMO volume (in water), Å^3^	surface electrostatic potential at C^4^	log*P*	preferred C^4^-pucker	amide bond		

						*K_trans_*_/_*_cis_* (in water, 25 °C)	*k_cis-to-trans_* (in water, 37 °C), s^−1^	*k_trans-to-cis_* (in water, 37 °C), s^−1^

Pro	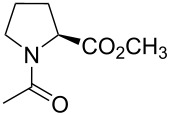	214	positive	−0.44 ± 0.05	*endo*/*exo* mix	4.95 ± 0.05	0.033 ± 0.002	0.0070 ± 0.0005
*R*-Flp	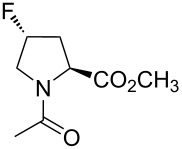	222	positive and negative	−0.66 ± 0.03	*exo*	7.16 ± 0.31	0.087 ± 0.009	0.012 ± 0.002
*S*-Flp	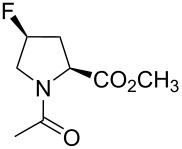	221	negative and positive	−0.84 ± 0.05	*endo*	2.62 ± 0.07	0.041 ± 0.004	0.016 ± 0.002
Dfp	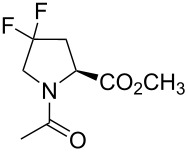	230	negative	−0.29 ± 0.04	*endo*/*exo* mix	3.49 ± 0.11	0.163 ± 0.016	0.049 ± 0.003

^a^See references [16,19,51–52,55] for details.

#### 2.1 Molecular size

**Figure 3:**
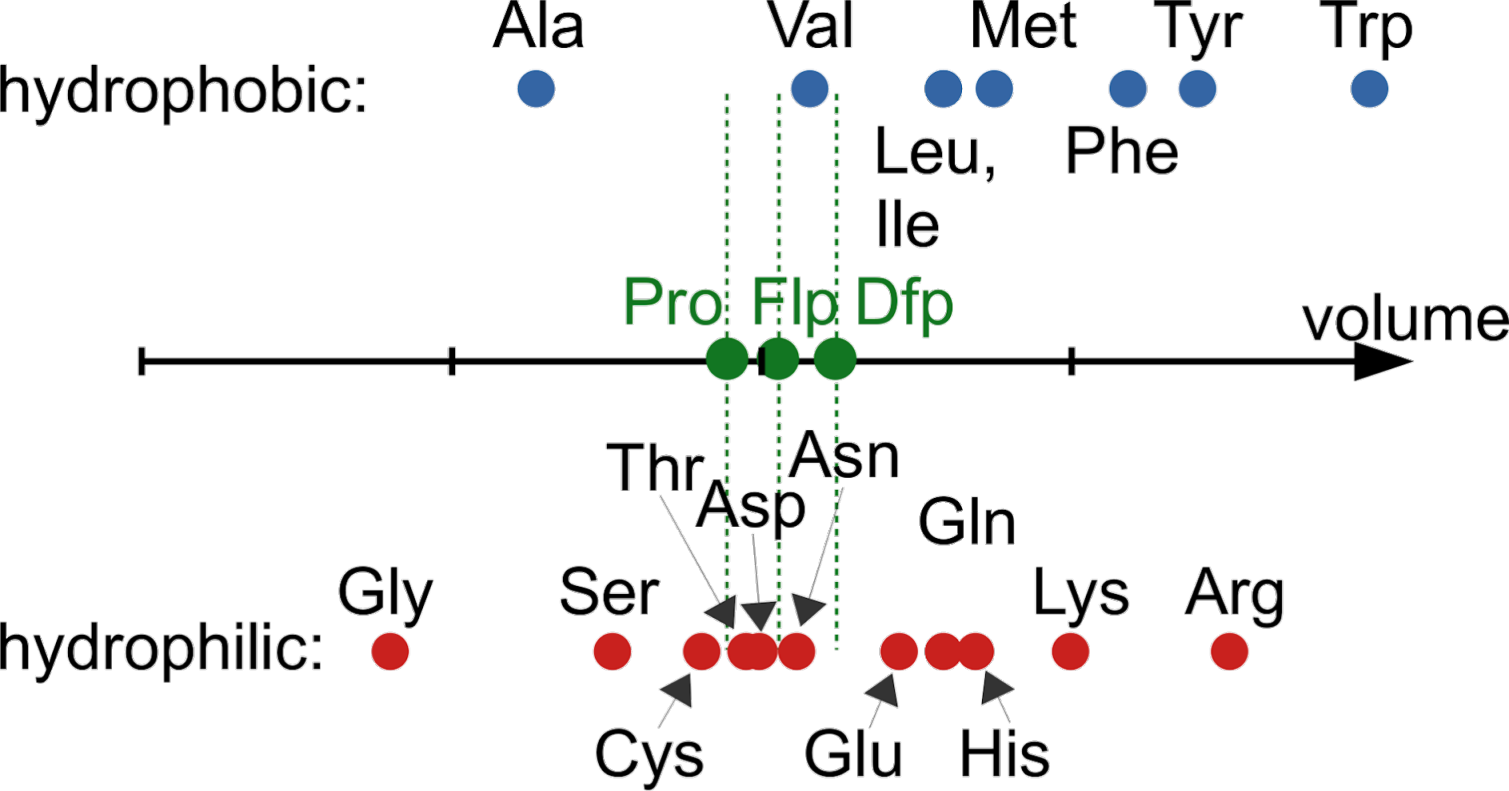
Molecular volume for 20 coded amino acids and fluoroprolines. The COSMO volume was calculated for aqueous solutions of free amino acids. The amino acids are classified as hydrophobic or hydrophilic according to their side chains. The scale is 50–250 Å^3^, with the tick step of 50 Å^3^.

Replacing hydrogen with fluorine increases the molecular size of congeneric molecules. The molecular volume increases gradually in the row: Pro < Flp < Dfp. However, compared to other amino acid size differences, this increase appears to be quite unspectacular (Figure 3). For example, the volume difference between Pro and Dfp is smaller than the difference between Val and Ile. It is therefore not surprising that Flp and Dfp can in principle be recognized as proline by natural enzyme binding pockets. The successful experimental incorporation of fluoroprolines into proteins based on the recognition of fluoroprolines by the native enzyme aminoacyl-tRNA synthetase (vide infra) proves this assumption.

#### 2.2 Local polarity

**Figure 4:**
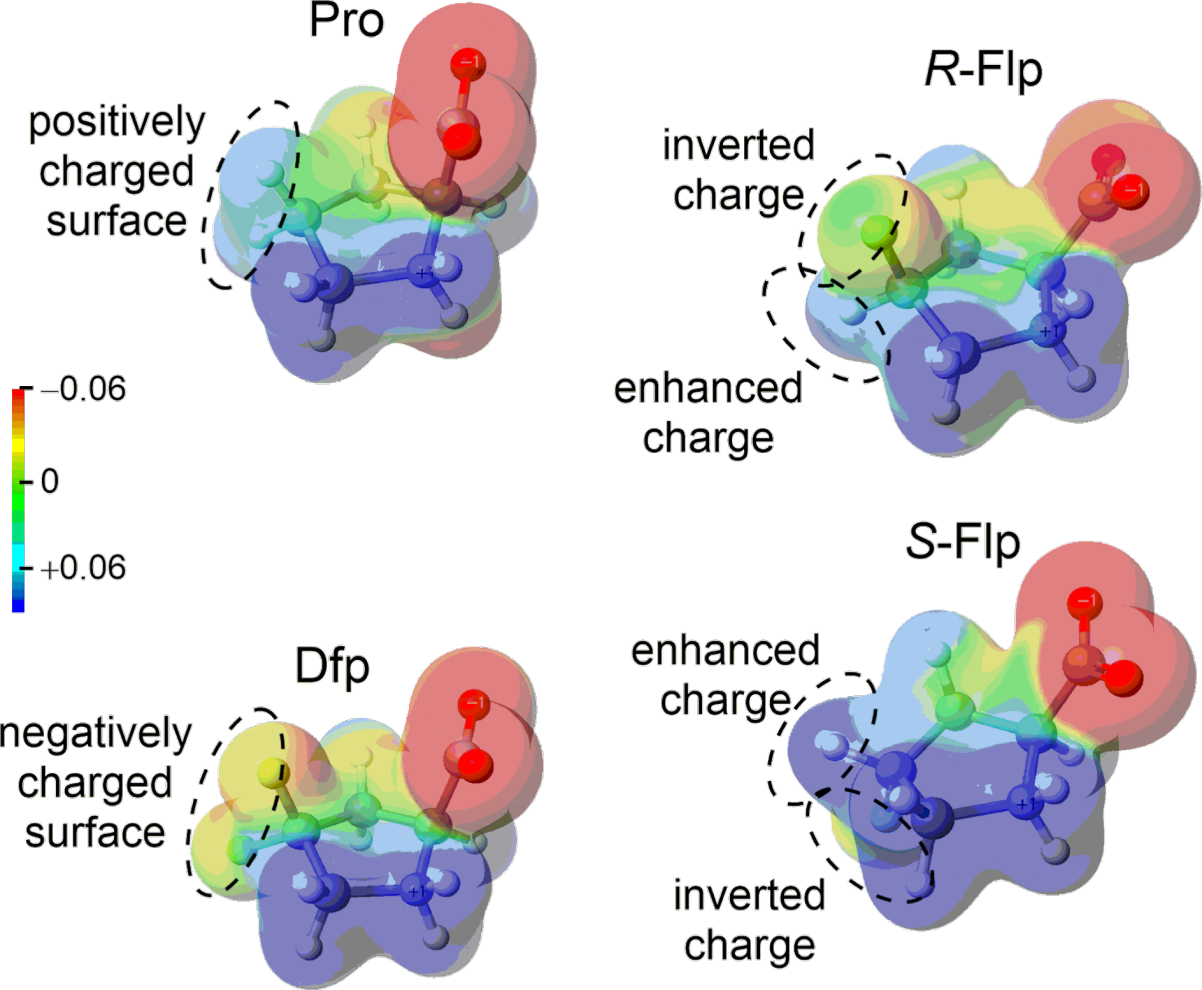
Comparative analysis of the electrostatic potential for proline and fluoroprolines (electrostatic potential on the van der Waals surface).

Another important feature of fluoroprolines is their local electrostatic potential (local polarity). In proline, position 4 contains a positive surface potential due to the hydrogen atoms at the exterior (Figure 4). This local charge plays an important role in the interaction of proline with the environment in a protein. For example, when a side chain of an aromatic residue docks onto the proline residue, they engage in a donor–acceptor interaction, while the aliphatic C–H bonds of the proline ring serve as electron acceptors. This phenomenon is known as the aromatic-proline motif [42–43]. The substitution of a hydrogen atom at position 4 by fluorine inverts the sign of the potential. The fluorine atom creates a surface that is negatively charged, while the remaining C–H bond in Flp acquires an enhanced positive charge due to the electron-withdrawing effect of fluorine. Based on these considerations, a fluoroproline–aromatic-system interaction has been previously utilized in the engineering of a few peptide therapeutics [44–45].

#### 2.3 Lipophilicity

**Figure 5:**
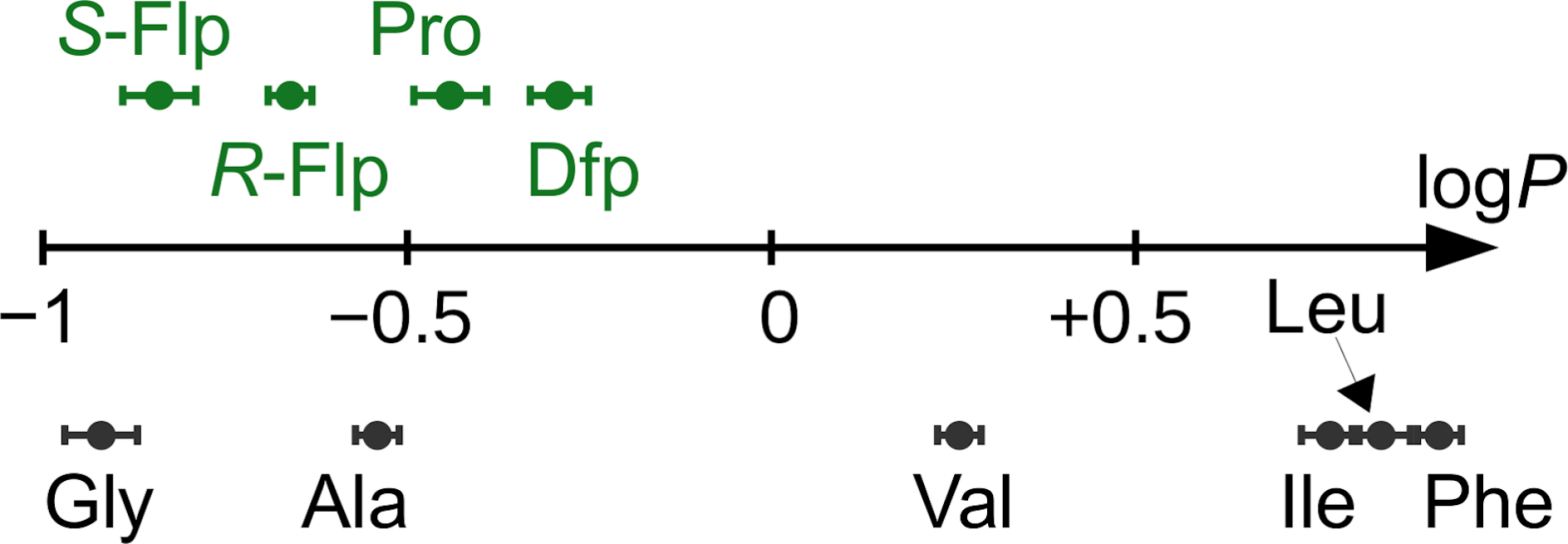
Experimental log*P* data for methyl esters of *N*-acetylamino acids.

The impact of fluorine substitution on the global polarity of the molecule is another parameter that should be considered. The global polarity can be typically analyzed by measurements of the molecular partitioning, e.g., the lipophilicity index log*P*. For aliphatic organofluorine compounds, this parameter takes into account the polarity of the carbon–fluorine bond, the combination of dipoles within the molecule, and the molecular volume [46–47]. The experimental log*P* values demonstrate that the monofluoroprolines *R*-Flp and *S*-Flp are slightly less lipophilic, while Dfp is slightly more lipophilic compared to proline (Figure 5) [19]. Although, the difference is small, it may have an impact on the overall features of the peptides and proteins that rely on polarity.

#### 2.4 Proline puckering

**Figure 6:**
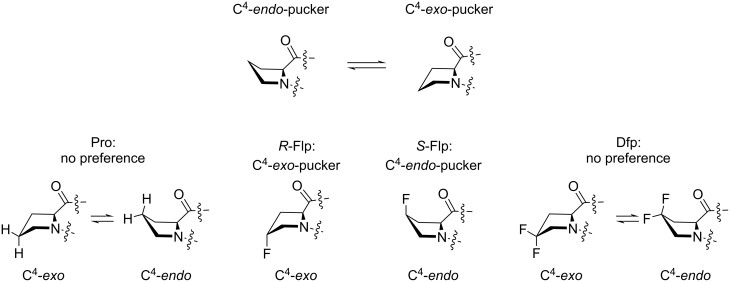
The conformational dependence of the proline ring on the fluorination at position 4.

In addition to the general impact of fluorination, there are some properties specifically attributed to the proline residue that appear altered in fluoroprolines. The first one to mention is the conformation of the pyrrolidine ring, which exhibits a dynamic transition between several distinct states in a relatively fast kinetic mode (roughly on a GHz scale) [48–49]. For simplicity, two conformations are considered most commonly, usually called C^4^-puckers. In the *endo*-pucker, the carbon atom C^4^ is displaced from the ring plane towards the carboxylate group, while in the *exo*-pucker, it is displaced in the opposite direction (Figure 6). Although, this may look like a mere side-chain conformation transition, this has a remarkable relevance. In principle, the whole pyrrolidine ring is conjunct with the backbone, and thus there is no conformational transition that would occur separately in the backbone and in the side chain; both should occur at the same time. In this way, the pucker influences the backbone folding, thereby stabilizing or destabilizing particular secondary structure folds.

Fluorination creates a pucker bias due to orbital interactions of the carbon–fluorine bond within the molecule, known as the *gauche*-effect [50]. The parent proline structure has a negligible energetic difference between the conformations. However, *R*-Flp stabilizes the C^4^-*exo*-pucker, while *S*-Flp stabilizes the C^4^-*endo* system [51]. It is believed that Dfp has no conformational preference akin to proline [52]. The stabilization of the puckers is one of the most prominent features of fluoroprolines that justifies their use in protein engineering. The "freezing" of certain proline pucker conformations due to stereospecific fluorination has an effect on the packing within a protein structure as well as the backbone folding parameters (vide infra).

#### 2.5 Thermodynamics of the amide rotation

**Figure 7:**
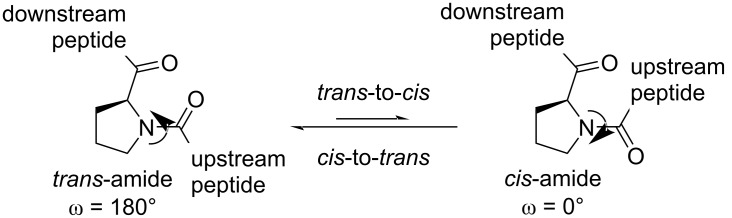
Rotation around the peptidyl-prolyl fragments in polypeptide structures is important for correct overall folding.

Another special feature of proline is the comparatively similar intrinsic stability of the amide rotamers in the peptidyl-prolyl fragments. Usually, a peptide bond exhibits a high preference towards the *trans*-amide, represented by a dihedral angle ω of 180°. In peptidyl-prolyl, however, *tran*s- and *cis*-conformations (ω = 180° and 0°, respectively) are of comparatively similar stability, while the *trans*-amide is more stable (Figure 7) [53]. In a folded protein, the overall conformation dictates the amide rotation state. Nonetheless, when the protein is unfolded, the *trans*/*cis* isomerism can create numerous subpopulations, and the resulting heterogeneity can markedly complicate folding. In addition, the stability of the amide bonds translates into the thermodynamic stability of the protein structures by altering the free energy of folding.

Fluoroprolines alter the relative thermodynamic stability of the amide conformers. Compared to proline, *R*-Flp provides a higher stability to the *trans*-conformer, while the *S*-Flp increases the stability of the *cis*-amide. The relative stabilization is usually about 1–2 kJ⋅mol^−1^ when measured for the typical model compounds. Dfp exhibits a relatively weak destabilization of the *trans*-amide [16,52]. These energetic preferences should be considered when judging the stability of the folded structures containing fluoroprolines.

#### 2.6 Kinetics of the amide rotation

The kinetic stability of the amide conformers generates another important aspect of protein folding. Generally, the amide rotation is considered very slow in biochemical settings [54]. The process becomes slightly faster when proline is replaced by fluorinated analogues. The acceleration occurs due to the electron-withdrawing effect of the fluorine atoms. According to the data for the model compounds in water [16,19], the *cis*-to-*trans* rotation velocity increases in the order Pro < *S*-Flp < *R*-Flp < Dfp. For the opposite process, the *trans*-to-*cis* rotation, the order is slightly different; Pro < *R*-Flp < *S*-Flp < Dfp (Figure 7). The process typically occurs in a mHz kinetic mode, as measured with the model compounds [16,19,55]. Environmental factors can change the velocity as well. For example, in the nonpolar environment of a protein interior or a hydrophobic pocket of a chaperone, the process may occur much faster [53]. Some protein sequences require the action of peptidyl-prolyl *cis*/*trans* isomerases, natural enzymes meant to facilitate the amide rotation around peptidyl-prolyl [56]. Some other common factors, such as the pH value or the ionic strength, are believed to have a very minor, if any, influence [53]. The enhancement of the amide rotation rate by fluoroprolines can have an effect on the overall protein folding velocity. An impairment of the folding kinetic pathways can also lead to aggregation and misfolding issues, affecting the protein production.

#### 2.7 Other properties

The acid–base transitions also appear altered in fluoroprolines primarily due to the electron-withdrawing effect of the fluorine atoms (Table 2) [16,19,55,57–58]. This effect may potentially influence the properties of proteins carrying terminal proline residues [59].

**Table 2:** Acid–base transitions in fluoroprolines.^a^

	ammonium moiety		carboxylic acid moiety			
		
			*cis*-amide conformation		*trans*-amide conformation	
entry	structure	p*K*_a_	structure	p*K*_a_	structure	p*K*_a_

Pro	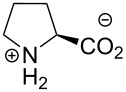	10.68	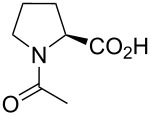	2.85	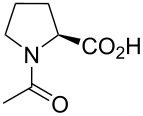	3.55
*R*-Flp	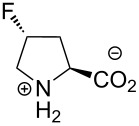	9.10	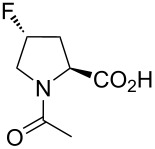	2.37	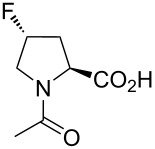	3.19
*S*-Flp	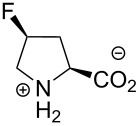	9.10	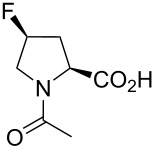	2.87	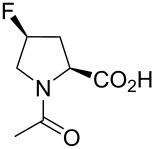	3.39
Dfp	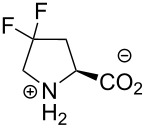	7.15	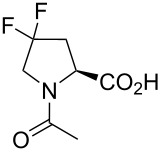	2.34	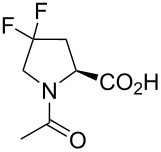	2.93

^a^Determined in aqueous solution at 298 K. The error for the p*K*_a_ value is ±0.10 for the ammonium group and ±0.05 for the carboxylic acid group. For details see references [16,19,57–58].

### 3 Proline uptake and metabolism

**Figure 8:**
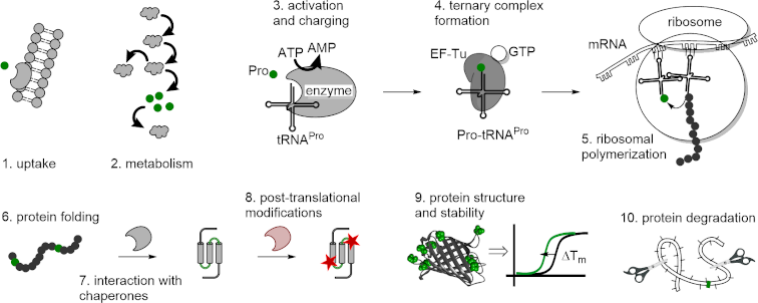
The complex fate of a protein-encoded amino acid in the cell (EF-Tu – elongation factor thermo unstable).

As the simplest and most basic set of structures, amino acids are involved in numerous biochemical processes in cells (Figure 8). The amino acid uptake and metabolism systems establish the concentration of the amino acid in the cells. The intracellular concentration enables the involvement of the amino acid in various cellular processes, and it reflects the cellular life cycle as well as the environmental perturbations, including starvation, oxygenation, osmolarity changes and other types of stress. For example, in exponentially growing *E. coli*, the intracellular concentration of proline was determined as 3.9 × 10^−4^ mol⋅L^−1^. This data ranks proline as the 41st in the list of metabolites and as the 9th among the amino acids coded into proteins [60].

The two main pathways for proline biosynthesis involve the synthesis from either glutamate or ornithine [61]. Both sources of amino acids are derived from the core metabolic processes. The connection to the central metabolism makes it difficult to make interventions in the production of proline in the cells. For example, in order to create an organism that fully lacks the ability to synthesize proline (which is called a proline auxotrophic strain), one should delete or inactivate genes involved in the metabolism of ornithine and glutamate. As the result, this may lead to an unnecessary accumulation of other amino acids and their metabolic derivatives, which may impact cellular homeostasis. In addition, proline metabolism is related to stress response and scavenging of reactive oxygen species in many organisms [62].

**Figure 9:**
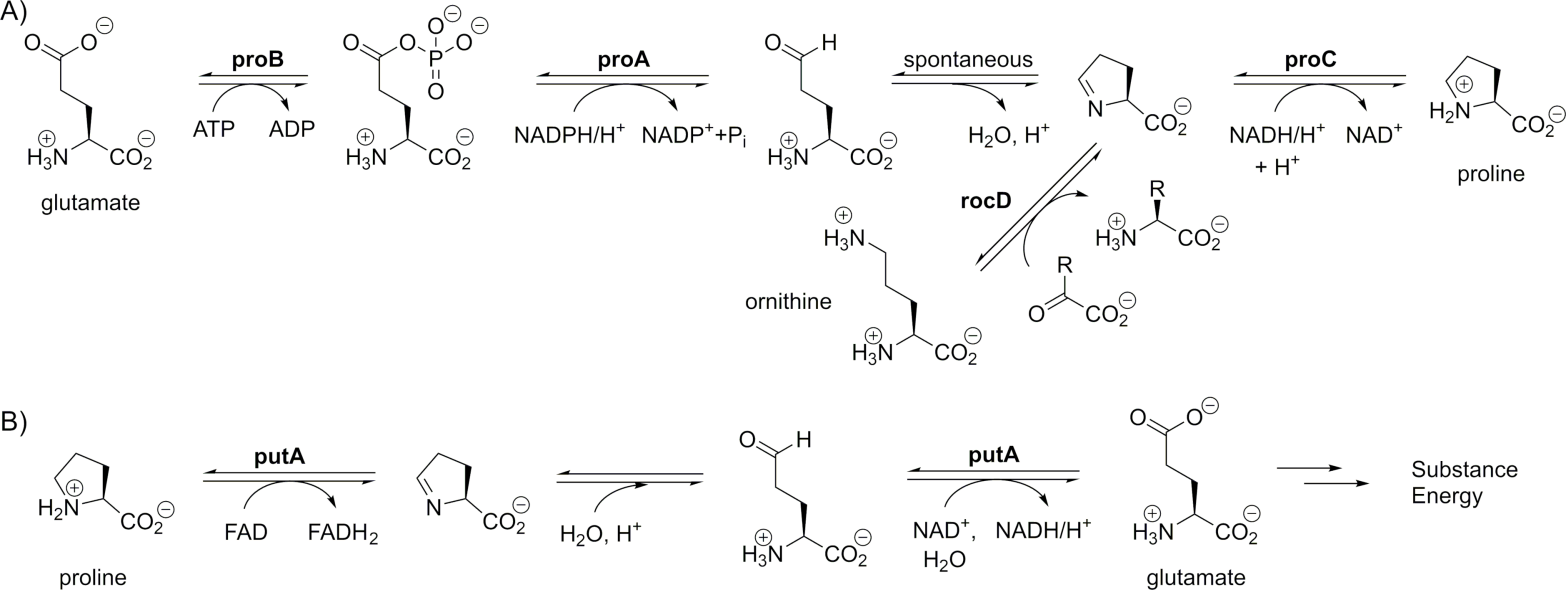
Metabolic routes for proline in *E. coli*. A) Synthesis of proline and B) degradation of proline.

In *E. coli*, the main path for proline synthesis starts from glutamate, which is phosphorylated by the action of glutamate 5-kinase (enzyme proB, Figure 9A). The resulting intermediate undergoes reduction into glutamate semialdehyde via the action of the γ-glutamyl phosphate reductase proA. The semialdehyde then undergoes spontaneous cyclization to form 1-pyrroline-5-carboxylate. In the final step, 1-pyrroline-5-carboxylate is reduced via the action of the pyrroline-5-carboxylate reductase proC, generating proline. Alternatively, glutamate semialdehyde can be created from ornithine via the action of a pyridoxal phosphate-dependent enzyme, the ornithine-oxo-acid aminotransferase rocD. Very recently, some fluorinated proline analogues were prepared via a chemoenzymatic transformation using the leucine hydroxylase griE [63–64].

In addition to the biosynthetic schemes, proline can be supplied to cells exogenously and taken up by specific permeases. Proline uptake serves two general functions: 1) the utilization of proline as a nutrient and 2) the uptake of proline in response to osmotic stress. The proline/sodium symporter putP is a transporter system that pumps proline into the cells, together with sodium ions. It is present in various organisms (including *E. coli*) and mainly supplies proline as an external nutrient [65]. Another important transport system is the ATP-binding proline/betaine transporter. In *E. coli*, this permease activates in the course of osmotic shock response, represented by the transporter gene *proP* and the *proU* operon. The genes *proV* (encoding an ATP-binding protein) and *proW* (encoding a permease) as well as *proX* (encoding a periplasmic protein) are located on the *proU* operon. The proline/betaine transporters accumulate proline and various other substrates inside the cells under high external salinity. They are tolerant to the transport of proline analogues, such as pipecolic acid [66].

In *E. coli*, the catabolism of proline occurs via the action of the bifunctional enzyme putA (Figure 9B) [67]. It sequentially degrades proline to glutamate, which can be later deaminated to an essential metabolite, α-ketoglutarate, with many metabolic options, such as an entry into the citric acid cycle. The dehydrogenation of proline is involved in numerous biochemical processes. For example, the dehydrogenation of proline linked to an acyl carrier protein makes a first step in the biosynthesis of some neurotoxins from cyanobacteria (*ana* gene cluster) [68]. The catabolic degradation of proline plays an important role in the development of cancer cells, making the inhibition of the proline dehydrogenase a promising method in cancer therapy [69–70]. There is no information on a possible intracellular degradation of fluoroprolines, although, they are included in some therapeutic dehydrogenase inhibition schemes [71]. Conversely, there is strong evidence that other proline analogues, dehydroproline and thiaproline, are oxidatively degraded in *E. coli* by the action of the bifunctional enzyme putA and the reductase proC [72]. Future research should examine whether fluoroprolines are metabolically inert substrates capable of accumulating at a high level in the cells without causing significant disturbances in the physiology of the host microbial organism.

The transport and metabolism of proline are important factors that should be considered in engineering bacterial strains capable of incorporating proline analogues into proteins. In order to express protein-containing fluoroprolines, one should take a strain that is unable to produce the original substrate–proline. Proline auxotrophic strains are typically produced by the deletion of the genes responsible for proline biosynthesis (Figure 9A). However, some strains, such as *E. coli* JM83, are known to be readily proline auxotrophic [73], and thus can be subjected for the expression of proteins with exogenously supplied fluoroprolines [74]. Experiments demonstrated that the intracellular accumulation of fluoroprolines from the medium can occur without additional manipulations. For some analogues, an osmotic shock triggers the uptake and accumulation of proline substitutes into the cells. Gruskin and co-workers successfully demonstrated the incorporation of hydroxyproline into recombinant proteins in proline auxotrophic *E. coli* in media with hyperosmotic sodium chloride concentrations [75]. To obtain high in vivo concentrations of the proline analogue, they took advantage of the phenomenon that proline and similar solutes actively accumulate in cells in response to the hyperosmotic shock in bacteria [76]. This enabled *E. coli* to accumulate proline substitutes in hyperosmotic media (e.g., with 0.6 M sodium chloride) and provided a sufficient intracellular concentration of the analogue loaded onto tRNA^Pro^ to support protein synthesis. The relevance of this scheme towards the intracellular accumulation of fluoroprolines still remains questionable.

### 4 Fluoroprolines in protein biosynthesis

In the genetic code, proline is assigned to four codons within the CC codon block: CCU, CCA, CCG and CCC. The entire genome of *E. coli* (strain MG1655) contains 59905 proline codons, whereby the CCC is rarely used (7401 occurrences), in contrast to most abundant CCG codons (31603 occurrences) [77].

**Figure 10:**
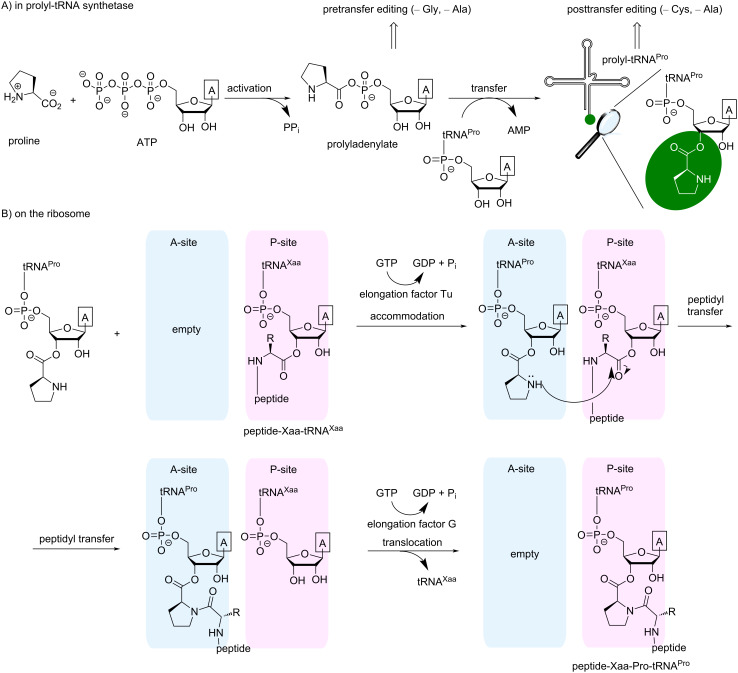
A complete flowchart for the proline incorporation into proteins during ribosomal biosynthesis. A) Activation and loading (charging) of an amino acid onto the tRNA^Pro^. B) The ribosomal cycle. A framed “A” identifies an adenine moiety. The A- and P-sites are the aminoacyl- and peptidyl-tRNA ribosome binding sites, respectively. The exit site (E-site) is not mentioned.

The incorporation of proline into proteins is accomplished by the natural biosynthetic machinery via a complex stepwise process (see steps 3–5 in Figure 8). The initial recognition of any amino acid that participates in protein biosynthesis occurs in an enzyme called aminoacyl-tRNA synthetase (AARS). An AARS usually performs several sequential steps (Figure 10A). First, an amino acid that fits into the binding pocket reacts with a molecule of ATP creating an aminoacyl adenylate. This step is called activation. An aminoacyl adenylate is a mixed anhydride, and therefore it is a highly reactive species, which quickly undergoes further transformation. In the subsequent step, the aminoacyl adenylate reacts with the CCA 3’-end element of bound tRNA, thereby creating an ester bond with the cognate tRNA. The *E. coli* genome contains three tRNA^Pro^ entities, having the anticodons CGG (recognizes CCG codon), GGG (recognizes CCC and CCU codons) and UGG (recognizes CCA, CCU and CCG codons). This set of tRNA^Pro^ exhibits an average level of charging compared to other tRNAs from *E. coli* [78].

Prolyl-tRNA synthetase (ProRS) belongs to a class IIa AARSs. In addition to the main activation and charging steps described above, the bacterial ProRS (gene *proS*) performs a few additional proofreading steps. Alanine and glycine are amino acids smaller than proline but with a similar polarity, and they can be activated by ProRS. If this happens, aminoacyl adenylate is released from the enzyme to be hydrolyzed by water. This process is called pretransfer editing. Cysteine is an amino acid that has a similar molecular volume to proline, and therefore cysteine can undergo activation and transfer by ProRS. To correct for this error, the transfer step is followed by the swinging of the charged tRNA to another pocket that is meant for the recognition of Cys-tRNA^Pro^ (the yeaK editing domain). If the amino acid is correct, the charged tRNA is released from the enzyme, and if cysteine is loaded on it, it is hydrolyzed. This process is called posttransfer editing step. There is also alanine posttransfer editing [79–80]. In *E. coli*, both pre- and posttransfer editing occur in ProRS. They only discriminate against substrates that are smaller than proline, whereas fluoroprolines are slightly larger than proline by volume (Figure 3). The ability of fluoroprolines to fit into the proline activation pocket and to avoid editing enables the use of natural ProRS for the incorporation of these substrates into proteins in place of proline. The enzyme accepts the substrate that is available; preferably proline, if it is present in the cells, and fluoroprolines once proline is depleted.

In general, amino acid analogues are less suitable for natural AARSs than their canonical substrates. Therefore, the intracellular concentration of an analogue should be high enough to promote the misacylation of cognate tRNA^Pro^. Another strategy towards the accumulation of the charged tRNA is to increase the genomic background activity of the ProRS by plasmid-directed coexpression of the native or mutant enzyme by a strong promoter during the incorporation experiment. Early experiments demonstrated sufficient activation of fluoroprolines by ProRS [81]. Conticello et al. reported that the native genomic background activity of ProRS was sufficient to load fluoroprolines to tRNA^Pro^ with a similar efficiency as proline [82]. A docking study showed that the binding of fluoroprolines to the ProRS from *E. coli* occurs in a similar fashion and with similar binding energy to that of the native substrate, proline [83].

After being released from ProRS, the charged Pro-tRNA^Pro^ binds to the elongation factor Tu, which also binds a molecule of guanosine triphosphate (GTP). The resulting complex (known as the ternary complex) docks to an empty A-site of a translating ribosome (Figure 10B). If the mRNA codon matches the anticodon of the tRNA^Pro^, GTP undergoes hydrolysis, allowing the elongation factor Tu to leave the complex. This event is followed by the peptidyl transfer reaction, which forms a new peptide bond. Subsequently, the ribosome binds the elongation factor G carrying a molecule of GTP, the hydrolysis of which leads to a translocation event in which the empty tRNA leaves and peptidyl-tRNA moves to the P-site. The A-site remains empty, allowing the next ternary complexes to bind in response to the next codon in the mRNA sequence [84].

**Figure 11:**
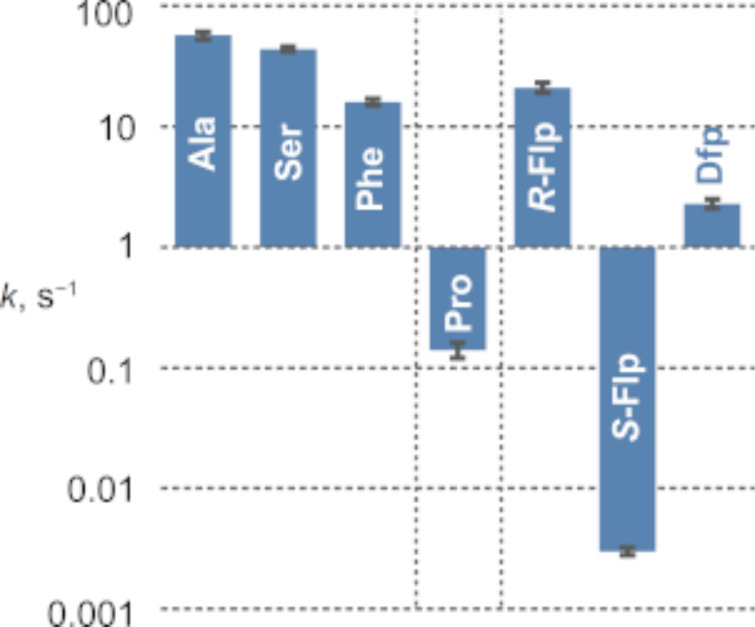
Amide bond formation capacities of fluoroprolines compared to some coded amino acids measured on ribosomes in an in vitro experimental setup. The rates of reaction between formyl-methionyl-aminoacyl-tRNA at the P-site and puromycin at the A-site of a translating ribosome (at 37 °C) were determined [57].

The translational incorporation of proline into proteins is a slow process when compared to other canonical substrates [85]. It has been shown that fluoroprolines, when used as proline substitutes, can substantially alter the velocity of translation. In a survey of proline analogues, it was found that *R*-Flp restores the reactivity of proline at the P-site to a level comparable to that of other coded amino acids (Figure 11) [57]. The enhancement of the translation velocity was also observed for Dfp. For *S*-Flp, however, the reaction was substantially slowed down even compared to the native substrate proline. The origin of this effect is not fully clear. It might be linked to the electrophilicity of the carboxylic acid moiety that experiences an electron-withdrawing effects from the fluorine atom (see the comparison of the acidity in Table 2) [58].

In another study, tRNA^Pro^ was preloaded with proline analogues, and a test sequence (formyl-Met-Lys-Lys-Lys-Xaa-Asp-Tyr-Lys-Asp-Asp-Asp-Asp-Lys-OH, Xaa = proline analogue) was expressed in an in vitro system. It was found that the yield of the target peptide increases in the order Pro ≈ *R*-Flp < *S*-Flp ≈ Dfp [86]. The origin of the effect is unclear. Nonetheless, these results show that *S*-Flp can actually produce better peptide yields compared to *R*-Flp, in spite of the poor translation velocity found in other experiments. Evidently, fluoroprolines are capable of producing spectacular changes in protein translation. Their elucidation might reveal unknown features of ribosomal translation that might be correlated to the physiochemical properties of fluoroprolines.

Where there are three or more consecutive prolines in the sequence, the ribosome stalls. The alleviation of the stalled ribosome occurs due to the action of elongation factor P, as was recently discovered independently by two groups [87–88]. Elongation factor P binds the exit site of the stalled ribosomes and recognizes specific identity elements of the tRNA^Pro^ at the P-site [89]. The overall effect of elongation factor P binding is that it restricts the set of conformations available in the peptidyl transfer center, and this suppresses the entropic costs of the reaction [57,90]. It has been suggested that ribosome stalling has a regulatory function in protein production as about one third of the *E. coli* genome contains stalling-inducing oligoproline stretches [91]. In general, the translation of oligoproline- and proline-rich sequences has to cope with the inefficient accommodation of prolyl-tRNA, a slowed rate of peptide bond formation at proline residues [85], and collisions between the nascent polyproline chain and the exit tunnel, in particular the residue G2061 [90].

We and others investigated whether ribosome stalling can be mitigated by proline analogues. For example, in the case of three consecutive *R*-Flp or Dfp residues, stalling was nearly absent in an in vitro experiment, while *S*-Flp exhibited stronger stalling compared to the natural substrate proline [57]. The strong stalling induced by *S*-Flp may preclude the production of proteins with this substrate as the in vivo expression of enzymes with oligoproline stretches might be completely suppressed with this analogue (vide infra). Chin and co-workers reported the directed evolution on ribosomes that yielded a variant (dubbed as O-d2d8) with mutations in the peptidyl transferase center and exit tunnel, able to efficiently polymerize proline-rich sequences in the absence of elongation factor P [92]. It is possible to presume that such ribosome variants could reduce *S*-Flp-induced ribosome stalling, although experimental demonstration is lacking. From the reported data, it follows that *S*-Flp may enhance ribosomal synthesis when it is a single residue in a sequence, while in the case of consecutive proline residues, *R*-Flp is a substitute that is expected for best production.

The folding of the peptide chain to secondary structure elements starts immediately after the synthesis, inside the ribosome tunnel. The tunnel accommodates between 30 and 40 amino acid residues [93–94]. The trigger factor (gene *tig*) is a generic chaperone that facilitates the folding of the N-terminal protein section emerging from the ribosome [95]. The proline residue is associated with a dedicated set of chaperones called peptidyl-prolyl *cis*/*trans* isomerases (PPIases), which correct the *cis*–*trans* rotameric state of proline in the protein sequence [53,96]. *E. coli* carries a diverse set of the chaperones of this class (e.g., the genes *ppiA*, *ppiB*, *fkpA*, *fklB* and more), including periplasmic chaperones (e.g., gene *surA*). A previous study showed that the presence of *S*-Flp, *R*-Flp and Dfp in a protein sequence may alter the chaperone-mediated folding efficiency by orders of magnitude [97]. The putative reason for this is the difference in the polarity of fluoroproline-containing sites, which alters the accommodation of the protein in the chaperone pockets. Furthermore, the proline-to-fluoroproline replacement may generate disturbances in the protein structure and stability, as discussed in the next sections.

### 5 The structural contexts of fluoroprolines in proteins

As discussed in chapter 1, proline is generally poorly compatible with the common secondary structure context of alanine-based residues. Nonetheless, there are specific structural motifs that are more common for proline residues, i.e., representing the “proline world”: the collagen helix, polyproline helix, turns, loops, and fragments lacking a persistent structure.

#### 5.1 Collagen triple helix

Many studies reported the role of proline-to-fluoroproline replacement in the collagen helix. The collagen triple helix is a super-secondary structure featured by collagen, the protein that constitutes the extracellular matrix in higher animals. When introduced at a certain position in the structure, *R*-Flp and *S*-Flp exhibit prominent effects on the thermal stability of the protein. The effect originates from the stabilization of the pyrrolidine ring puckers, which has an effect on the packing of the triple helix [98]. The reader is referred to dedicated reviews on collagen model studies that discuss the impact of fluoroprolines in more detail [35,99].

#### 5.2 Polyproline-II helix

The polyproline-II (P_II_) helix is a secondary structure that precedes the collagen helix in the folding hierarchy. It is a left-handed extended helix that attained its name from the original discovery in polymeric proline. The P_II_ helix occurs in various proteins, and it is particularly common in proline-rich sequence fragments [100]. Studies of oligomeric peptides showed that the replacement of proline with *R*-Flp exerts a stabilizing effect on the P_II_ helix formation, while *S*-Flp has an opposite, destabilizing effect [101–102].

Proline-rich sequences are common in SH3 binding domains of proteins. A study identified a minimal binding motif (peptide Pro-Pro-Pro-Leu-Pro-Pro-Lys-Pro-Lys-Phe) and demonstrated that the recognition occurs when the peptide is structured in the form of a P_II_ helix [103]. In spite of this fact, the replacement of proline with the P_II_-stabilizing analogue *R*-Flp failed to improve the binding to SH3 [104–105]. These observations indicated that the polyproline binding is not only sensitive to the secondary structure adopted by the substrate, but an altered polarity and steric features of fluoroprolines should play a role.

Our recent study included the incorporation of fluoroprolines into the bacteriophage T4 fibritin C-terminal domain (foldon). The results showed that *S*-Flp and *R*-Flp exhibited opposite stability effects when incorporated at the positions Pro-4 and Pro-7, despite the fact that both residues are located in the N-terminal P_II_ helix. This result was interpreted by differences in the local polar interactions created by the carbon–fluorine bond within the interior of the foldon domain [106].

#### 5.3 Turns and loops

Another structural context common for proline residues is turns. A peptidyl-prolyl in the *cis*-amide conformation is essential for the formation of type-VI β-turns [107]. Other types of β-turns also favor the presence of proline. A study of a model peptide (acetyl-Xaa-Gly-NHCH_3_, Xaa = proline analogue) showed that type-I β-turns tolerate favored pucker conformations of both *R*-Flp and *S*-Flp. In contrast, type-II β-turns prefer the formation of the C^4^-*exo*-pucker in both *R*-Flp and *S*-Flp residues, and thereby type-II β-turns experience stability changes from the intrinsic pucker biases exerted by fluoroprolines [108]. As a result, fluoroprolines could significantly impact the transition temperature in elastin-like sequences [108–109]. In ligand-gated ion channels, proline residues are involved in numerous loop positions as well as in the transmembrane helices, and the incorporation of fluoroprolines has shown distinct effects on their activity [110–113].

#### 5.4 Intrinsically disordered proteins

Proline residues are overrepresented in proteins that are lacking persistent structures, known as intrinsically disordered proteins [114]. Here, proline acts with a dual role: 1) as a breaker, proline precludes the formation of regular secondary-structure motifs and 2) as a conformationally constrained residue, proline nucleates the formation of structures needed for binding with other proteins [115–116]. The potential role of fluoroprolines on the structural and functional features of intrinsically disordered proteins remains unclear.

### 6 Fluoroprolines in crystal structures of proteins

**Figure 12:**
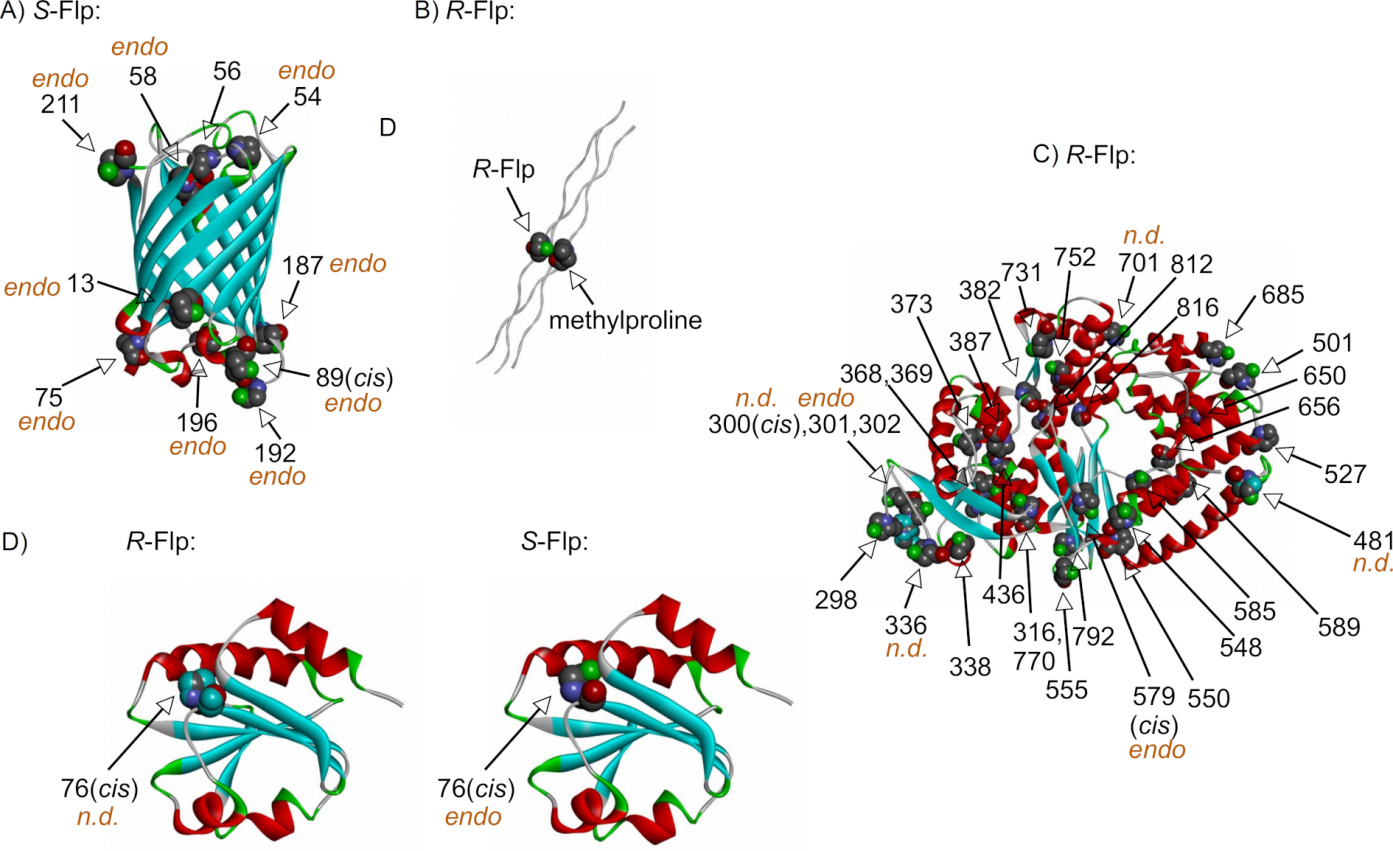
Ribbon representation of the X-ray crystal structures of proteins containing fluoroprolines. A) Enhanced green fluorescent protein (pdb: 2q6p), B) collagen mimicking model (pdb: 3ipn), only one fluoroproline residue is shown, C) KlenTag DNA polymerase (pdb: 4dle) and D) thioredoxin A (pdb: 4hua and 4hu9). All C^4^-*endo*- and undefined puckers are labeled accordingly, assuming that the rest of the residues adopt the *exo*-pucker conformation. All *cis*-amide positions are labeled as well, assuming the default arrangement being *trans*.

Several high-resolution crystal structures have been reported for proteins containing fluoroprolines. In a study of enhanced green fluorescent protein from *Aquorea victoria*, fluoroprolines were incorporated at ten proline positions [117]. The incorporation of *R*-Flp resulted in an insoluble protein, indicating associated folding issues. In contrast, the protein containing *S*-Flp produced a well-structured fluorescent protein, exhibiting faster folding kinetics and producing crystals suitable for diffraction. The structure (Figure 12A) displayed ten *S*-Flp residues: nine with a *trans*-amide and one with a *cis*-amide (position 89). With the exception of one residue (position 56), all pyrrolidine rings were found in a C^4^-*endo*-pucker conformation. This result is consistent with the intrinsic *endo*-pucker bias of *S*-Flp (Figure 6), which appears reproduced in the complex protein structure.

A study of collagen mimetics demonstrated an elevated thermal stability for the collagen helix formed by repeating *R*-Flp-Gly-*S*-Mep units (where *S*-Mep is (4*S*)-methylproline) [118]. The collagen triple helix was found in the crystal structure of the protein (all residues were in the *trans*-amide conformation). The structure showed the *R*-Flp and *S*-Mep residues adopting *exo*- and *endo*-pucker conformations, respectively. This arrangement created a tight packing between the *R*-Flp and *S*-Mep side-chains, as shown in Figure 12B. The packing effect has an implication on the thermostability of the triple helix [35,98–99].

In another study, fluoroprolines were incorporated in KlenTag DNA polymerase from a thermophilic organism, *Thermus aquaticus* [119]. The expression of the target protein was only observed with *R*-Flp, but not with *S*-Flp. The protein containing *R*-Flp exhibited a slightly lowered stability compared to the parent enzyme. The crystal structure depicts *R*-Flp at 32 different positions (Figure 12C), including two proline residues forming *cis*-amide bonds (positions 300 and 579) and two oligoproline stretches (positions 300–302 and 368–369). A defined pucker could be observed clearly in 28 out of 32 residues; 26 residues out of this set were in the *exo*-pucker conformation. In contrast, the crystal structure of the wild-type protein showed 18 residues adopting single pucker conformations, and only 7 from this set were in the *exo*-pucker conformation. This observation showed that the preference of the *R*-Flp to adopt a C^4^-*exo*-pucker conformation is retained in the protein structure. A defined side-chain conformation of the *R*-Flp-containing protein was proposed to be the reason for the ease of the protein crystallization. Finally, the study revealed a few polar interactions of the fluoropyrrolidine ring within the protein microenvironment.

In a study on thioredoxin, four out of five proline residues were mutated to alanine, and the only remaining proline residue was the one adopting a *cis*-amide-bond conformation (position 76) [120]. Both *S*-Flp and *R*-Flp were incorporated into the mutant. The resulting protein variant showed an elevated stability in the reduced, and a lowered stability in the oxidized form of the protein. Crystal structures of the proteins containing Pro, *S*-Flp and *R*-Flp at position 76 demonstrated their high similarity (Figure 12D). The variants containing Pro and *R*-Flp did not show a clearly defined pucker, whereas the variant with *S*-Flp was found in a single C^4^-*endo*-pucker conformation.

Overall, the crystal structures illustrate that the pucker preference observed in small molecules (Figure 6) reoccurs in the protein structures, given that a statistically significant number of residues is analyzed. Nonetheless, in some individual cases, the native preference of a fluoroproline residue can be overridden due to the residue microenvironment and the overall packing of the protein structure.

### 7 The stability of proteins containing fluoroprolines

In the context of biochemical science, it is common to assume that structural changes and stability differences correlate with each other. For a protein, an overall sum of the secondary structures can be easily evaluated by chiroptic methods, such as circular dichroism, which assesses the backbone folding [121–122]. Changes in the denaturing conditions can be tracked by circular dichroism or fluorescence-based assays very easily. Alterations in the regular fold would usually be associated with alterations in the energy of the folding. These observations created a common conception that structure and stability experience the same trends. The less the structure is preserved, the less stable the folding of the protein is, and vice versa.

However, in proteins with fluorinated amino acids, structure and stability do not necessarily correlate, as demonstrated by studies on aromatic amino acids [123]. In fact, the survey of the crystal structures presented in chapter 6 demonstrates that the native backbone fold of the protein can remain unaltered, while associated biochemical assays may show notable differences in the thermodynamic stability of the fluoroproline-containing proteins. How could it happen that the stability is affected but the structure is not? First of all, in contract to classical mutations (e.g., the replacement of proline with serine), a substitution of an amino acid by a fluorinated analogue generates very little disturbance to the structure itself ("atomic mutations"). At the same time, it alters the conformational and polar features of the residues. For example, for fluoroprolines, there is an adaptive dynamic of the pucker in the pyrrolidine ring. One could expect, for instance, that a replacement amino acid would have to adopt an unfavorable conformation in order to fit to the overall structure (e.g., an *exo*-pucker conformation for *S*-Flp). Under these circumstances, the entire protein structure may be formed as expected; however, the replacement amino acid would strain the whole structure. Such a scenario would result in a lowering of the melting point and an elevated susceptibility of the structure towards denaturing agents. Thus, the stability will be impacted, while structure remains intact. For that reason, these two properties should be assessed separately when discussing fluoroprolines as well as other fluorinated amino acid substitutions [124–126].

As a matter of fact, most of the literature dealing with hydrogen-to-fluorine atomic mutations in proteins reports changes in the stability and not in the structure. Stability changes have been reported for barstar [16,127], tryptophan cage miniprotein [128], the villin headpiece subdomain [129], *T. thermohydrosulfuricus* lipase [130–132], human ubiquitin [133], single-chain Fv format protein [134], KlenTag DNA polymerase [135], thioredoxin A [120], β2-microglobulin [136], red fluorescent protein [137–138], Pin1 WW domain [139], bacteriophage T4 fibritin C-terminal domain [106] and ribonuclease A [140] (Table 3). These results have been explained by considering the conformational bias of the residues (pucker, *trans****/****cis* equilibrium), an altered hydrophobicity and polarity and the local environment at the substitution sites. An elevated folding velocity, presumable due to faster amide rotation kinetics, has been reported for fluoroproline containing cysteine-rich minicollagen domains [141], green fluorescent protein [117,142], ubiquitin [133], β2-microglobulin [136], thioredoxin A [119,143] and red fluorescent protein [137]. In addition, the effects of the fluoroproline incorporation on the enzymatic performance were characterized for a few enzymes [59,130–132,135] and the activity of ion channels [110–113]. These studies generally showed the preservation of the fold of the parent enzymes inferred from the preservation of their native activities.

**Table 3:** Stability changes in proteins and protein domains produced upon proline-to-fluoroproline replacements.^a^

protein		number of replaced proline residues		change in the thermodynamic stability			references
				*R*-Flp	*S*-Flp	Dfp	

barstar		1		↓	↑	≈	[16,127]
tryptophan cage miniprotein		1		↑	↓	not examined	[128]
enhanced green fluorescent protein		10		insoluble	folded, no data	not examined	[117,142]
villin headpiece subdomain		1		↓	≈	not examined	[129]
*T. thermohydrosulfuricus* lipase		6		↓	↓	not examined	[130–132]
human ubiquitin		3		↑	no production	not examined	[133]
single chain Fv format protein		8		↑	insoluble	not examined	[134]
KlenTag DNA polymerase		32		↓	no production	not examined	[119,135]
thioredoxin A single proline	reduced	1		↑	↑	not examined	[120,143]
	oxidized	1		↓	↓		
β2-microglobulin		1		↓	↑	↓	[136]
bacteriophage T4 fibritin C‐terminal domain		2	position 4 position 7	↓ ↑	↑ ↓	not examined	[106]
red fluorescent protein (mRFP1)		12 (11)		↑	insoluble	not examined	[137–138]
Pin1 WW domain		1		↓	↑	not examined	[139]
ribonuclease A		1		not examined	↑	not examined	[140]

^a^As summarized from the thermal stability and other reported data that reflect the stability.

From these data, it could be deduced that fluoroprolines generally cause minimal perturbations in protein structures. This hypothesis is indeed supported by the wealth of available protein studies. However, such a conclusion would be premature. We would like to recapitulate at this point that a study of a protein relies on its production, which is mainly achieved via living cells. If fluoroprolines alter the protein folding, a misfolded sample would usually be prone to aggregation and/or degradation by the cellular machinery. This may be seen as a lack of protein production or as the protein being insoluble. Only proteins that maintain the overall folding will be properly produced, isolated and characterized in a biochemistry laboratory. This requirement creates a natural bias in the biochemical literature, which largely ignores so-called “negative results”. A misfolded protein is a typical negative result, which is usually neglected for further investigations. Structural disturbances are also not welcome in spectroscopic studies of fluorine-labeled proteins by ^19^F NMR spectroscopy. Effectively, it is much better and easier to study a protein that is properly folded, rather than a protein that is misfolded. This is why the structural changes cannot be ruled out, despite the fact that no such instance has been documented in the literature. They may be “hiding” among the proteins that demonstrate a lack of expression or proteins that showed incoherent laboratory results and were therefore never recorded in the scientific correspondence.

**Figure 13:**
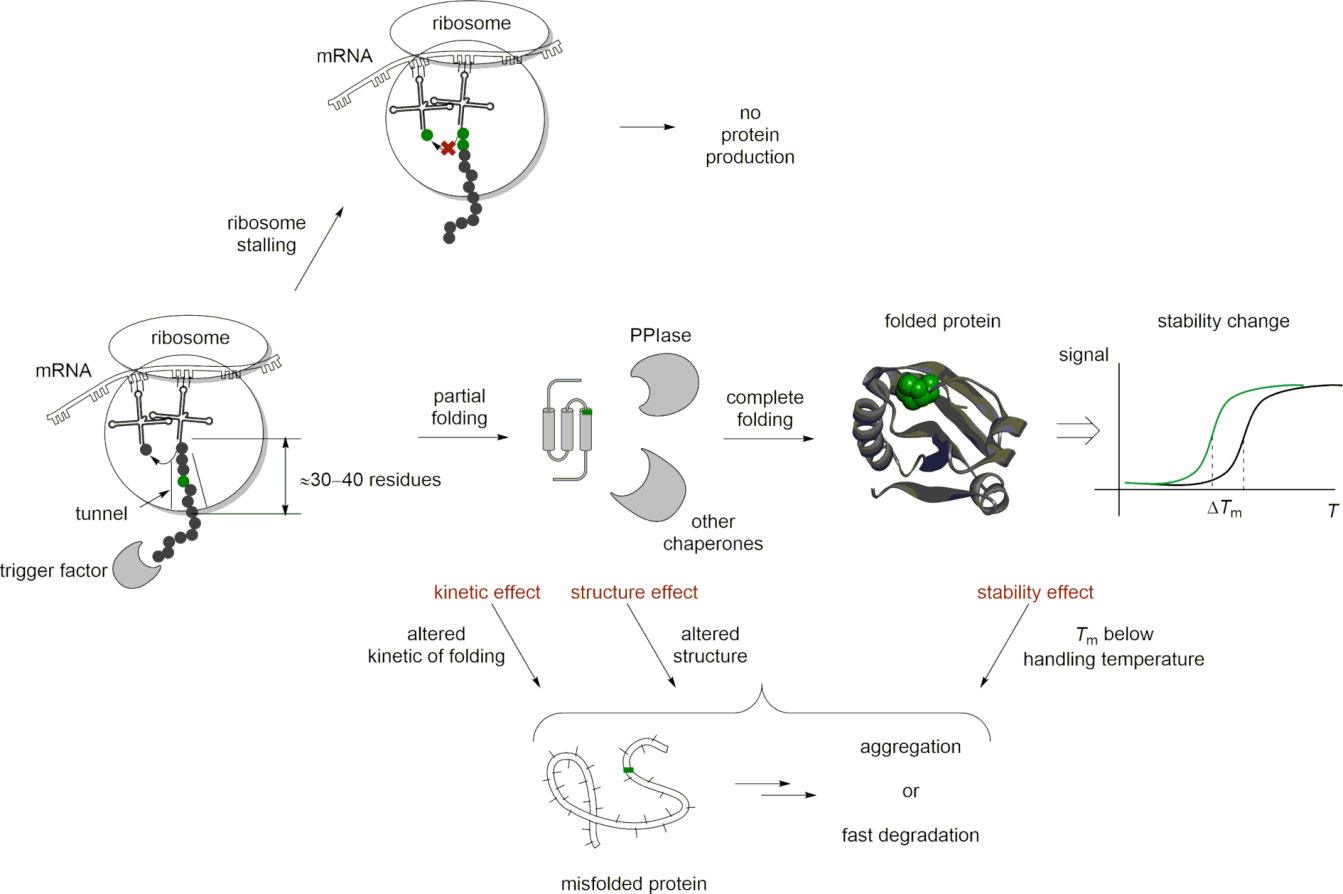
Problems and phenomena associated with the production of a protein-containing proline-to-fluoroproline substitution.

The failure to express a certain protein carrying the proline-to-fluoroproline substitution is not uncommon in laboratory practice. It can be caused by various factors: 1) an improper fermentation setup, 2) a lack of translation (e.g., due to the ribosome stalling with *S*-Flp), 3) a lack of interaction with chaperones, 4) misfolding that leads to aggregation, 5) an inhibitory effect of the target protein on the cellular machinery and 6) misfolding that leads to rapid degradation of the protein, to name a few. A misfolding can occur for reasons such as an altered structure, incorrect velocity of the folding steps, and lowering the stability of the protein below handling temperature (Figure 13). In contrast, a folded protein can be produced, isolated and characterized.

**Figure 14:**
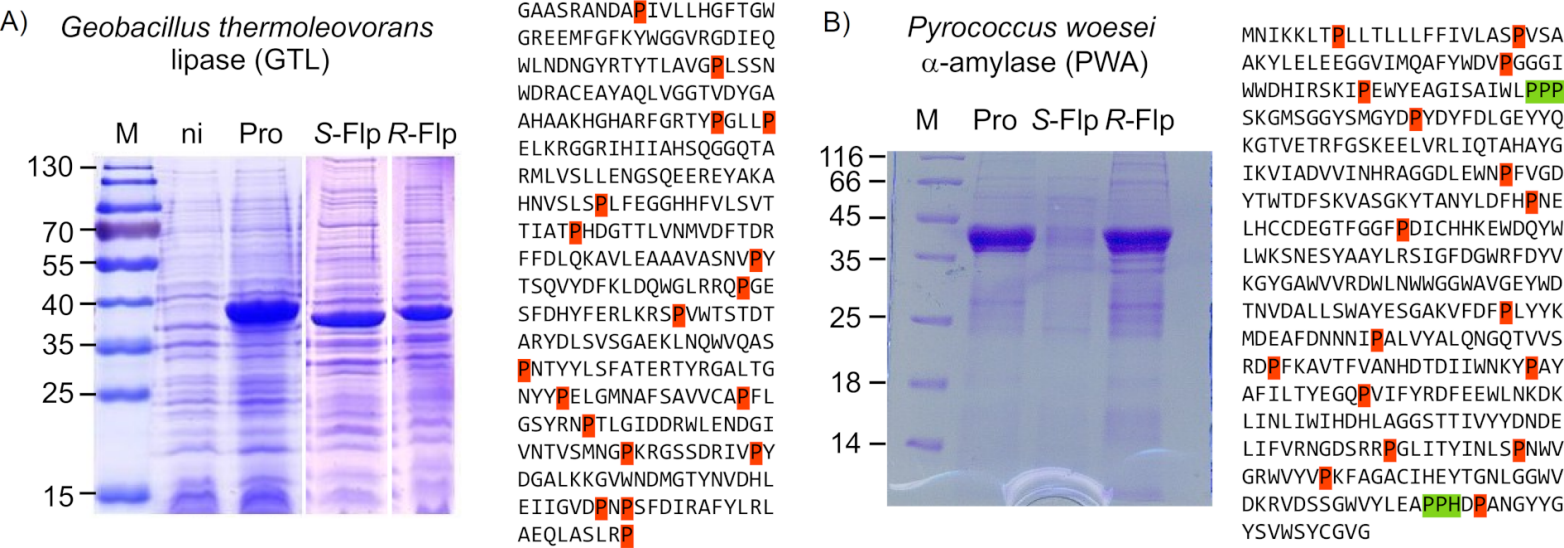
Effects of fluoroprolines on recombinant protein expression using the auxotrophic expression host *Escherichia coli* JM83. Left: sodium dodecyl sulfate–polyacrylamide gel electrophoresis expression profiles. Right: sequence compositions of corresponding proteins with marked single proline residues (orange) and oligoproline stretches (green). ni – non-induced (control) cell fraction, M – molecular weight (kDa) marker. A) *Geobacillus thermoleovorans* lipase (GTL) with 18 individual proline residues distributed throughout the protein sequence is expressed in the insoluble (inclusion bodies) form in the presence of *S*-Flp (Federica Agostini, unpublished results). B) In the presence of *S*-Flp in the growth medium, no expression of α-amylase from *Pyrococcus woesei* (PWA) could be detected (Michael Hoesl, unpublished results). Note, that in addition to 17 individual proline residues in the sequence, PWA also contains two additional oligoproline stretches. However, expressions in the presence of *R-*Flp yielded soluble and active variants for both enzymes.

Figure 14 illustrates the expression of two proteins with fluoroprolines. The expression of *Geobacillus thermoleovorans* lipase (GTL) yielded a properly folded sample with *R*-Flp and an insoluble protein with *S*-Flp (Figure 14A). The lack of solubility in the latter case indicates critical folding issues induced by the presence of *S*-Flp in the sequence. With α-amylase from *Pyrococcus woesei* (PWA), the expression experiment in the presence of *S*-Flp failed to produce the full-length protein (Figure 14B). The interpretation for this fact is the lack of protein expression due to the ribosome stalling induced by the oligoproline stretches in the sequence of the target protein. These two examples illustrate a diversity of complications that could hinder protein studies with fluoroprolines.

On the other hand, in the presence of *R*-Flp, the folded and fully active PWA enzyme was isolated, clearly demonstrating the ability of this analog to abolish the ribosome stalling issues in vivo. This is in full agreement with the in vitro results, showing attenuation of the stalling in oligoproline stretches with three consecutive *R*-Flp residues in the absence of elongation factor P [57]. Therefore, the use of fluoroprolines could be considered as an interesting biotechnological strategy and opportunity to optimize the expression of proteins and enzymes of interest by circumventing translational issues.

The final aspect of the protein chemistry is protein degradation. Aliphatic fluorinated amino acids have been utilized towards an enhanced proteolytic stability of peptides [144]. The information regarding the impact of fluoroprolines in peptide and protein degradation is lacking though.

### 8 The potency of computational modeling in the design of proteins containing fluoroprolines

The conformational consequences of the fluorination of the 4-position of proline have been well studied using computational methods. The latter provided extensive documentation of the electrostatic interactions within the molecules and the observed conformational biases [145–146]. These studies have been further extended to examine the influence of neighbouring amino acids and short peptide chains [108]. However, there has been little done to explore the energetic significance of the conformational preferences and interactions of fluoroprolines in folded proteins [147]. Early work using molecular dynamics has explored differences in the ring pucker conformation and the interactions of fluoroprolines with water molecules in collagenous structures. Yet, there is still much left unexplored in this area, including models examining the enhanced stability of proteins containing fluoroprolines. This is particularly important since the systematic incorporation of fluoroprolines and other proline-based derivatives will facilitate the encoded cellular synthesis of biopolymers with a nonnatural backbone chemistry.

The lack of computational models is likely due to limited structural and stability data on fluoroproline-containing peptides. As the field continues to grow and in-depth characterization of these newly designed fluoroprolines becomes available, so too can the accuracy of computational models used to evaluate the effects of fluoroprolines on protein stability. Several factors that influence the conformational preferences of fluoroprolines in a folded protein have already been identified, indicating that intermolecular forces may override the inherent preferences for ring pucker (see chapter 6). To better understand the roles of stereoelectronic factors and intermolecular forces in dictating the ring conformation and protein stability, future research must include detailed systematic studies involving site-selective mutations of fluoroproline. From these studies, the impact of individual fluoroproline residues in a protein can be assessed, and factors involved in an increased stability can be assessed using molecular dynamics and quantum mechanics/molecular mechanics studies. The results of this future work would help to identify key features that need to be considered when designing a fluoroproline-containing protein: which sequence positions are most critical to enhance the protein stability, and which diastereomer of fluoroproline to introduce into the protein sequence to obtain a desired effect.

### 9 Towards living cells relying on the supplement of fluoroprolines

**Figure 15:**
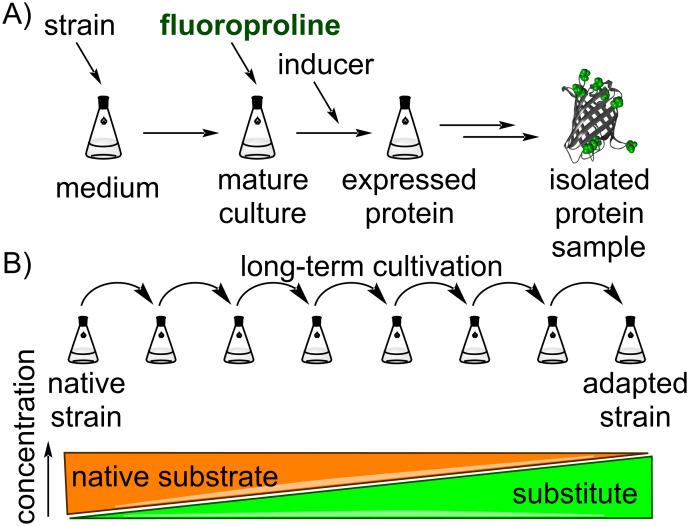
A) Experimental setup for the incorporation of fluoroprolines into proteins. B) Adaptive laboratory evolution (ALE) experiment for the replacement of a canonical substrate with an artificial substitute.

The success of the incorporation of fluoroprolines into proteins by “hijacking” the native *E. coli* machinery suggests a potential of proteome-wide substitution with these proline analogues. Indeed, if fluoroprolines can substitute proline in a single protein, why not substitute proline in a whole cell? Instrumentally, the production of a protein containing a fluoroproline can be done in a relatively easy manner (Figure 15A). First, a proline auxotrophic strain should be enaculated in a medium under a limited proline concentration, which does not allow the culture to grow fully to the stationary phase point. After the proline in the medium is used up, the fluoroprolines should be added, and the the target protein gene is induced, leading to a production of the protein of interest. In this way, the cell machinery is forced to accept fluoroproline for the protein polymerization due to the lack of proline in the medium. Such machination is often referred to as selective pressure, thus the method is called selective pressure incorporation. It leads to production of a protein, where all proline residues are subjected for replacement with the substitute, thus the incorporation is global. Although efficient in production of single proteins, this method makes use of the native microbial culture, which remains unaltered in this approach.

Conversely, the introduction of fluoroprolines into the proteome of a living organism implies alteration of the entire proteome of the cell. Potentially, such an intrusion could be addressed by following the general strategies known as bottom-up and top-down [148]. The bottom-up approach implies the recreation of a living cell from the first principles, while in the top-down approach, an already existing complex living organism should be redesigned [149–150]. The bottom-up approach could be exemplified as the synthetic recreation of compartments that bear functions of organelles or living cells.

Efforts have been directed towards a bacterial adaptation to tryptophan-to-fluorotryptophan replacement via the engineering of the corresponding AARS [151–152]. In another approach, an adaptation of *E. coli* to fluorotryptophan was successfully performed using adaptive laboratory evolution (ALE) [153–154]. In ALE, the driving force for the adaptation was created by the gradual replacement of indole (tryptophan) by its fluorinated analogue, while the bacterial strain was not able to produce its own tryptophan via the native cellular machinery. The cells first had to allow the diffusion or uptake of fluoroindole, followed by a simple metabolic conversion to fluorotryptophan, which serves as a replacement for all tryptophan residues in the proteome. Since the cells are dependent on the presence of tryptophan in the growth medium, fluorotryptophan was used in this experimental setup as a nutrient essential for growth. The selective pressure to adapt cells to the fluorinated tryptophans was thus achieved by combining metabolically modified cells (auxotrophs) with the externally created artificial conditions, i.e., a defined growth medium with a controlled chemical composition. (Figure 15B).

The replacement of proline by fluoroprolines could be attempted instrumentally in a similar fashion. Nonetheless, in contrast to tryptophan, this replacement may be facilitated by the beneficial molecular effects of fluoroprolines in protein chemistry. For example, high-resolution X-ray structures showed that the enhanced green fluorescent protein (Figure 12A) with its β-barrel structures contains proline residues with pyrrolidine rings exhibiting predominantly C^4^-*endo*-puckers. It is reasonable to expect that the incorporation of *S*-Flp, which favors *endo*-puckers, will further increase the refolding capacity of these proteins, while *R*-Flp-containing protein will be irreversibly unfolded after expression (including purification and refolding attempts). On the other hand, some proteins, such as the KlenTag DNA polymerase (Figure 12C), perform better when containing *R*-Flp as the proline substitute. Although the biochemistry of fluoroproline incorporation still did not provide a critical mass of experimental evidence for generalizations, it is not difficult to imagine the scenario in which the adaptation of, e.g., auxotrophic *E. coli* strains to grow in the exclusive presence of fluoroproline might only work with a certain mixture of both diastereomers, instead of the analogues being added to the culture substrate individually. The hypothetical adaptation of the bacterium or other cells to one of the fluoroproline residues would certainly be an enormous breakthrough, as it would not only require the reconfiguration of numerous metabolic and regulatory networks but also an accompanying co- and posttranslational folding of the resulting polypeptides.

By performing experiments to achieve the proteome-wide insertion of fluoroprolines into microbial cells, we should be able to answer the question to what extent naturally evolved protein scaffolds and related cellular machineries and systems are suitable for the accommodation and integration of these isosteric building blocks. The adaptation of bacteria to fluorinated tryptophans discussed above is rather the exception, since tryptophan is a rare amino acid (only 20688 codons in the *E. coli* genome [77]), and hydrogen-to-fluorine replacement on the indole side-chains represent "atomic mutations", which are known to be well tolerated even in investigated isolated proteins [123–124,155] and proteomes [153–154]. Thus, it should be taken into account that the natural structural scaffolds with hydrocarbon cores have been formed and optimized through billions of years of evolution and may not be suitable to accommodate a large number of fluorine atoms [1,8]. We have shown this in our previous experiments on the global substitution of methionine (37698 codons) and leucine residues (144466 codons) in cellular proteins by related trifluoromethylated analogues [156]. These experimental attempts lead to the conclusion that the inclusion of such a fluorine-containing building block would require a novel repackaging of the protein core [132].

While trifluoromethylated analogues of coded amino acids (such as Val, Leu, Ile, Met or some aromatic side-chains) are simply too bulky, steric bulk should not be the main barrier to the accommodation of fluoroprolines in proteins and proteomes (see chapter 2.1). Nevertheless, the incorporation of fluoroprolines into the proteome changes the backbone features of each individual protein and subsequently could lead to a large number of misfolded proteins promoting the expression or down- and upregulation of genes encoding chaperones and proteases. Next, it may induce the stress response that normally redirects energy resources and regulatory networks otherwise dedicated to growth. To overcome such an unpleasant scenario, cells will only survive if they are able to evolve appropriate "coping" mechanisms, allowing them to grow continuously in the presence of fluoroprolines.

This scenario should create the selective pressure as the fundamental basis of adaptation experiments of cells and may lead to the accumulation of random mutations in their genomes. Cellular phenotypes with a suitable set of mutations that favor the use of fluoroproline, which should also provide them with a fitness gain over the generations in a given environment (medium). In other words, these cells with suitable mutations, accompanied by reconfigured metabolic, lipid composition, signaling and transcription networks, should proliferate and assert themselves in the culture medium faster than the others. This could be a possible evolutionary scenario to convert fluoroprolines from stressors to proteinogenic amino acids that support cell growth, i.e., essential nutrients.

Once this goal has been achieved, one could further evolve the fluoroproline-dependent organism towards a state where proline is no longer accepted as the protein constituent. Nonetheless, at the present state of enzyme engineering, it is not possible to envision how a fluoroproline-accepting-proline-rejecting enzymatic mechanism could be developed as the molecular volume and polarity are very similar for these amino acids. Perhaps, the simultaneous engineering of the activation pocket of ProRS towards fluoroprolines as slightly larger substrates, and editing the pockets towards proline as a slightly smaller substrate could lead to the creation of a dedicated fluoroprolyl-tRNA synthetase. However, it is still unclear how to achieve such a sophisticated engineering or how to introduce this manipulation into a living organism without immediate lethal effects for the host organism.

Finally, in order to achieve a true "fluorine life" with a top-down approach, the metabolism itself must be reengineered or created de novo to ensure a continuous supply of the fluorine-containing metabolic blocks. Furthermore, structural and kinetic readjustment of otherwise highly conserved translation factors and the achievements of the novel quality control of fluoroproline-based protein translation should reshape the mechanism behind the flow of genetic information, especially if xeno nucleic acids are included in this process [157–158]. We are only at the beginning of a long journey. For the time being, it remains to be seen whether fluoroprolines could be a suitable vehicle to take us beyond the horizons of the “alanine world” towards the novel “proline world” [25].

## Conclusion

Despite the seemingly simple structure, proline is a peculiar amino acid involved in numerous cellular processes. The unique features created by proline residues in proteins justify the essential role of proline in biochemistry. Moreover, our recent theory suggests that proline was one of the first amino acids recruited for protein translation [22]. This suggests that the proteome originally evolved around proline as its universal constituent, which makes the proteome-wide replacement of proline a challenging task.

Fluoroprolines might be excellent candidates to address this challenge. These proline analogues create minimal perturbations in the volume and polarity of the parent proline residue. At the same time, their conformational effects alter protein production and folding. The enhancement of the protein stability, facilitated interaction with chaperones, elevated folding velocities, the mitigation of ribosome stalling – each of these effects known for fluoroprolines can create an advantage for cell fitness and survival, thereby forming a driving force for the adaptation for proline-to-fluoroproline replacement. At present, it is still unclear to what extent fluoroprolines could fulfill all functional tasks of proline residues. While favoring one of the C^4^-puckers and *cis*- or *trans*-amide bonds, fluoroprolines disfavor the others. The negative surface potential created by the fluorine atom and the dipole of the carbon–fluorine bond can cause additional problems for the packing inside the protein interior, which originally evolved to accommodate proline residues. At the same time, many details on the interaction of fluoroprolines with the cellular machinery are still missing, and they should be addressed by future research.

Seminal experiments on the adaptation of the whole cell to fluoroprolines are expected in the nearest future. They should provide novel information on the basic chemical makeup of life and create a path towards artificial biodiversity that involves fluorine as a bioelement.
